# Mobile Application Applied for Cognitive Rehabilitation: A Systematic Review

**DOI:** 10.3390/life14070891

**Published:** 2024-07-18

**Authors:** Su-Min Cha

**Affiliations:** Department of Occupational Therapy, Kyungnam University, Changwon 51767, Republic of Korea; csm1206@kyungnam.ac.kr; Tel.: +82-055-249-6479

**Keywords:** mobile application, cognitive rehabilitation, cognitive disorder, cognitive function

## Abstract

The COVID-19 pandemic has increased the demand for home rehabilitation services, leading to the development and rising demand for cognitive rehabilitation apps. However, a comprehensive analysis of the content and effectiveness of these apps is needed. This study systematically reviewed and analyzed the literature on mobile apps for cognitive rehabilitation. One researcher and an external expert conducted the study selection, data extraction, and quality assessment following the PRISMA Checklist 2020 guidelines. Our review of 18 studies identified 8 randomized controlled trials (RCTs) (44.44%) of high quality and 10 non-RCT studies (55.56%) of moderate quality. Overall, 16 of the 18 studies (88.88%) demonstrated positive clinical implications for cognitive function after using cognitive rehabilitation apps. Eight studies showed a correlation between app use and improved cognitive function, and four highlighted the potential for cognitive function evaluation through apps. All studies employed various occupational therapy (OT) intervention approaches, with prevention being the most common (100%), followed by establishment and restoration (83.33%), maintenance (77.77%), and creation and promotion (38.88%). This study clinically validates the effectiveness of cognitive rehabilitation mobile applications from an occupational therapy perspective.

## 1. Introduction

As society ages, the prevalence of geriatric diseases, such as dementia, mild cognitive impairment, and stroke, is increasing. Consequently, the number of people with cognitive disabilities is steadily rising [1,2,3]. Many researchers have emphasized the importance of cognitive rehabilitation in reducing behavioral and cognitive consequences and promoting independence and quality of life [4,5,6]. The need to adapt the healthcare system to meet these emerging needs and ensure continuity of care is evident [7]. Moreover, during the COVID-19 pandemic, the potential of using innovative technologies to provide healthcare services at home has been emphasized [8,9,10]. Technology’s ubiquity, even among older adults, has led to the development of numerous smartphone and web-based applications (apps) designed to assist individuals with mild cognitive impairment (MCI). Within cognitive training and stimulation, studies have identified promising strategies for preserving cognitive function in healthy older adults and individuals with MCI [11]. Previous studies on cognitive training game applications have shown that smartphone game applications can diagnose, prevent, and alleviate dementia [12] and that cognitive stimulation-based applications have been applied to the elderly to prove their effectiveness in enhancing cognitive function [13].

Recently, digital therapeutics using software such as mobile applications, games, virtual reality, and artificial intelligence have gained popularity [14], including in the field of cognitive training [15]. Despite the growing popularity of mobile apps for cognitive training, there remains a need for more evidence regarding their effectiveness as digital therapeutics. Previous studies have analyzed the quality of mobile apps for conditions such as low back pain [16] and speech disorders [17]. However, there is currently a lack of comprehensive analysis regarding the content and effect of cognitive training apps. Various mobile apps have recently been developed, but research is essential to verifying the effectiveness of cognitive training most suitable for different population groups and cognitive domains. These challenges can be addressed by verifying and comparing the efficacy of various approaches, such as computer-based training, treatment using gamified mobile apps, and the application of mobile apps combined with physical training [18].

This study systematically assessed the literature on mobile applications for cognitive rehabilitation and analyzed their effectiveness from an occupational therapy perspective. We hope to gather data that will aid in developing and applying cognitive rehabilitation-based applications to prevent and treat cognitive disorders. We expect this study to provide valuable guidance to researchers developing cognitive training apps, cognitively disabled individuals, and medical professionals selecting effective cognitive training apps.

## 2. Materials and Methods

### 2.1. Study Design

Our organized literature review on mobile applications for cognitive rehabilitation was conducted in accordance with the comprehensive PRISMA Checklist 2020 [19], instilling confidence in the thoroughness of our study design. We identified and categorized pertinent studies, compiled the results, and identified gaps in the field.

### 2.2. Eligibility Criteria for Study Inclusion

The PICO framework established eligibility criteria for study inclusion, ensuring the inclusivity of our review. Studies were considered if they met the criteria, allowing for a comprehensive examination of the field.

(1) Participants: Studies included participants with cognitive impairment due to nervous system disorders such as dementia, stroke, and traumatic brain injury, including mild cognitive impairment. It also included healthy subjects to prevent cognitive impairment.
(2) Interventions: Studies that applied interventions for cognitive function rehabilitation (evaluation, training, education, guidance, adaptation, prevention, etc.) and interventions based on portable mobile apps such as mobile phones and tablet PCs.
(3) Comparison: We compared the types of mobile apps, occupational therapy intervention approaches, and cognition training subdomains, as well as the types of cognitive disorders experienced by individuals with these disorders and healthy controls. We also incorporated descriptive research (i.e., with no comparison group).
(4) Outcomes: Studies that measured the cognitive effects of mobile apps for cognitive rehabilitation.

The exclusion criteria were as follows:

(1) Studies involving subjects whose main symptoms are mental illness (major depressive disorder, schizophrenia, etc.) or behavioral disorder (psychotic symptoms, mood disorders, nervousness, apathy, or other behavioral symptoms).
(2) Studies that applied interventions for rehabilitation purposes, such as physical function, sensory function, and language function, excluded cognitive function and did not include cognitive function in the outcome measurement.
(3) Studies on applying computer-based interventions using only the equipment in designated places (usually hospitals, daycare centers, institutions, etc.).
(4) Unrelated or redundant studies.
(5) Studies in which the original text is unavailable or only abstracts are presented.
(6) Non-original articles, such as review studies, letters, study protocols, poster presentations, books, etc.
(7) Non-English language articles.

### 2.3. Search Strategy

Our search strategy, conducted through June 2022 and in line with the Cochrane Handbook for Systematic Reviews of Interventions, ensured we found the most relevant publications. We identified relevant studies published between January 2010 and June 2022 in the Medline, Embase, and Cochrane Central Register of Controlled Trials (CENTRAL) databases, using three clusters of applied keywords (cognition disorder, mobile application-based cognitive rehabilitation, and cognitive function) to guide our search (Table 1).

### 2.4. Study Selection

One researcher and one external expert conducted the study selection, data extraction, and quality assessment for this study. Two reviewers evaluated each of the selected studies according to the search procedure. Initially, the studies were selected based on the title and abstract according to the criteria presented in the PICO section. Disagreements about inclusion were resolved by consensus. The two reviewers then evaluated the full-text version of each included study and decided whether to include it in the review. Again, disagreements about inclusion were resolved by consensus.

Two reviewers who used the search method as their guide rated each of the chosen studies. The title and abstract were initially considered for selection based on the PICO section’s criteria. A consensus was reached to settle differences in inclusion. The two reviewers examined the full-text versions of all the included studies and then decided whether to include them in the review. Once more, consensus overcame differences regarding inclusion.

### 2.5. Data Extraction Process

First, two reviewers performed data analysis, assessing the relevance of the review concerning the study questions and objectives. This was based on information from each study’s title, abstract, and keywords. The following data were gathered from each study on a Microsoft Excel 365 spreadsheet: title, authors, year of publication, journal, country of the study, study design, type of intervention, subject characteristics (mean age, sex ratio), session frequency and duration, outcome measurement, results, effectiveness, name of the app, type of platform, type of occupational therapy intervention approach, and subdomain of cognition. Afterward, all information was analyzed and summarized (Table 2, Table 3, Table 4, Table 5 and Table 6). Due to the non-randomized controlled trials extracted in this study, a meta-analysis was not performed.

### 2.6. Study Quality Assessment

One researcher and one external expert conducted a qualitative evaluation of 18 studies in this study. The Physiotherapy Evidence Database (PEDro) scale and the Methodological Index for Nonrandomized Studies (MINORS) were applied to evaluate the qualitative level of the selected studies. The PEDro scale was used for randomized controlled trial studies, and the MINORS was used for nonrandomized controlled trial studies. Two reviewers assessed the evidence level of each qualitative evaluation tool. If the opinions of the two reviewers differed during the qualitative evaluation process, the final score was calculated by reaching an agreement through discussion. The PEDro scale is a valid measure of the methodological quality of clinical trials. It responds with ‘Yes’ or ‘No’, and the maximum score is 10 points (the number of ‘Yes’). A qualitative evaluation from a methodological perspective is conducted with 9–10 points being ‘excellent’, 6–8 points being ‘good’, 4–5 points being ‘fair’, and 4 points or less being ‘poor’ [20]. We assessed the methodological quality of the retrospective articles using the MINORS score, with a global ideal score of 16 for non-comparative studies and 24 for comparative studies [21]. We then determined the percentage of quality criteria met, i.e., below 25% (very low methodological quality), 25–49% (low quality), 50–74% (moderate quality), and above 75% (high quality). This method of categorizing continuous criteria has been previously reported [22].

## 3. Results

### 3.1. Literature Search and Selection

**Figure 1:**
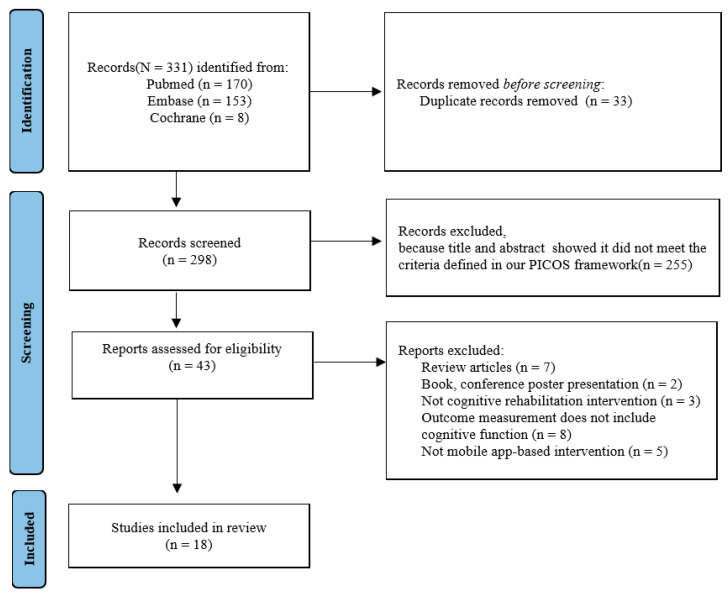
The PRISMA flow chart shows details of the processes related to the identification, screening, and selection processes. PICO = Participants, Interventions, Comparisons, Outcomes, Study design.

The initial search retrieved a total of 331 studies. After excluding 33 duplicate studies, the remaining studies were evaluated based on the inclusion criteria. Consequently, 255 studies were excluded, and following the full-text evaluation of 43 studies, a further 25 studies were excluded. Finally, 18 studies were included in this study (Figure 1).

### 3.2. Study Quality Assessment

The PEDro scale was used for eight randomized controlled trial studies, and the MINORS scale was used for ten nonrandomized controlled trial studies. The PEDro scale scores of the eight studies ranged from five points (fair) to nine points (excellent). Seven of these studies, excluding one, demonstrated good or higher quality (Table 2). The MINORS scores of the ten studies ranged from 9 to 12 points out of a maximum of 16 points and from 15 to 18 points out of a maximum of 24 points. Most of the studies showed moderate to high quality (Table 3).

**Table 2:** PEDro scale score.

	Corbett et al. (2015) [23]	Pedullà et al. (2016) [24]	Oh et al. (2018) [25]	Elbogen et al. (2019) [26]	Moon and Park (2020) [27]	Robert et al. (2020) [28]	Meltzer et al. (2021) [29]	Scullin et al. (2021) [30]
1. Eligibility criteria	Y	Y	Y	Y	Y	Y	Y	Y
2. Randomization	Y	Y	Y	Y	Y	Y	Y	Y
3. Hidden assignment	Y	Y	N	Y	Y	N	N	N
4. Group homogenous	Y	Y	Y	N	Y	Y	N	Y
5. Subjects blinded	Y	Y	N	Y	N	N	N	Y
6. Therapists blinded	N	N	N	N	Y	N	N	N
7. Assessors blinded	N	Y	N	N	Y	Y	N	N
8. Follow-up subjects	N	Y	Y	N	N	Y	Y	Y
9. Intention to treat	Y	Y	Y	Y	N	Y	Y	Y
10. Comparisons between groups	Y	Y	Y	Y	Y	Y	Y	Y
11. Scoring and variability measures	Y	Y	Y	Y	Y	Y	Y	Y
Total score	7	9	6	6	7	7	5	7
Quality criteria	Good	Excellent	Good	Good	Good	Good	Fair	Good

**Table 3:** MINORS score.

	Oliveira et al. (2014) [31]	Zygouris et al. (2017) [32]	Bonnechère et al. (2018) [33]	Powell et al. (2017) [34]	Chudoba et al. (2020) [35]	Tsoy et al. (2020) [36]	Oirschot et al. (2020) [37]	Bonnechère et al. (2021) [38]	Rosenblum et al. (2021) [39]	Weizenbaum et al. (2021) [40]
1. A clearly stated aim	2	2	2	2	2	2	2	2	2	2
2. Inclusion of consecutive patients	2	1	2	2	1	2	1	0	1	1
3. Prospective collection of data	2	2	1	0	1	2	2	0	2	2
4. Endpoints appropriate to the aim of the study	2	2	2	2	2	2	2	2	2	2
5. Unbiased assessment of the study endpoint	0	0	0	0	0	1	0	2	0	0
6. A follow-up period appropriate to the aim of the study	0	1	1	1	2	1	1	2	1	1
7. Loss of follow up less than 5%	0	2	2	2	2	1	2	0	0	1
8. Prospective calculation of the study size	0	0	0	1	0	1	0	1	1	0
Item 9–12 only for comparative studies										
9. An additional control group	2	2	1	1	-	-	2	-	-	-
10. Contemporary Groups	1	2	1	1	-	-	2	-	-	-
11. Baseline equivalence of groups	2	2	2	2	-	-	2	-	-	-
12. Adequate statistical analyses	2	2	2	2	-	-	2	-	-	-
Total score/Max. possible score	15/24	18/24	15/24	16/24	10/16	12/16	18/24	9/16	9/16	9/16
Quality criteria	Moderate	High	Moderate	Moderate	Moderate	High	High	Moderate	Moderate	Moderate

### 3.3. General Characteristics of Studies

The general characteristics of the studies were summarized by author, year, title, journal name, research country, research design, target group, sample size, average age of the study subjects, and gender ratio. The data presented in Table 4 are summarized in the order of the oldest publication. Of the eighteen studies, fourteen (77.77%) were conducted over the past six years [25,26,27,28,29,30,33,34,35,36,37,38,39,40]. Two studies (11.11%) were published in the Journal of Alzheimer’s Disease [32,33], while the remaining sixteen (88.89%) were published in different journals. Regarding the study countries, the USA had the most studies, with six (33.33%) [26,30,34,35,36,40], followed by the UK [23,38] and South Korea [25,27] with two studies each (11.11%). Portugal [31], Italy [24], Greece [32], Belgium [33], France [28], the Netherlands [37], Canada [29], and Israel [39] each had one study (5.55%).

The study design included eight randomized controlled trials (44.44%) [23,24,25,26,27,28,29,30] and ten nonrandomized controlled trials (55.56%) [31,32,33,34,35,36,37,38,39,40]. The subtypes of the nonrandomized controlled trials were as follows: non-equivalent control pre–posttest design [31,34] and cohort study [32,40] with two each; cross-sectional design [33,37] and case study design [35,39] with two each; and one-group pre–posttest design [36,38] with one each. The target group included three studies [23,29,38] on healthy individuals, with sample sizes larger than those of other studies, such as 12,000 [38] and 6742 [23] subjects. The remaining studies involved 3 to 104 subjects with cognitive impairments due to stroke, traumatic brain injury (TBI), mild cognitive impairment (MCI), multiple sclerosis (MS), and Parkinson’s disease (PD). Regarding the age of the study subjects, there was one study on young adults in their 20s [33] as a control group and studies on young adults in their 30s [26,35] as an experimental group, but most studies were conducted on older adults (between their forties and eighties).

**Table 4:** General characteristics of studies.

No.	Author (Year)	Title	Journal	Country of the Study	Study Design	Target Group	Sample Size	Age, M ± SD	Sex, Male/Female (%)
1	Oliveira et al. (2014) [31]	Cognitive Assessment of Stroke Patients with Mobile Apps: A Controlled Study	*Studies in Health Technology and Informatics*	Portugal	Nonequivalent control group pretest–posttest design	Stroke patients	*N* = 30 stroke patients (*n* = 15) healthy control subjects (*n* = 15)	Stroke patients: 45.5 ± 12.3 Healthy control subjects: unknown (age and education-matched to the stroke patients)	Stroke patients: 9(60)/6(40) Healthy control: Unknown
2	Corbett et al. (2015) [23]	The Effect of an Online Cognitive Training Package in Healthy Older Adults: An Online Randomized Controlled Trial	*Journal of the American Medical Directors Association*	United Kingdom	Randomized controlled trial	Healthy participants aged over 50 years old	*N* = 6742 Reasoning and problem-solving cognitive training (ReaCT) (*n* = 2557) General cognitive training (GCT) (*n* = 2432) Control (*n* = 1753)	ReaCT: 58.5 ± 6.5 GCT: 59.1 ± 6.4 Control: 59.1 ± 6.6	ReaCT: 805(31.5)/1752(68.5) GCT:754(31.0)/1676(68.9) 2 missing (0.1) Control: 660(37.6)/1093(62.4)
3	Pedullà et al. (2016) [24]	Adaptive vs. non-adaptive cognitive training by means of a personalized App: A randomized trial in people with multiple sclerosis	*Journal of Neuro Engineering and Rehabilitation*	Italy	Randomized controlled trial	Multiple sclerosis with cognitive impairment	*N* = 28 [Experimental Group (EG), Control Group (CG): Unknown]	EG: 49.0 ± 7.1 CG: 46.1 ± 11.2	8(28.6)/20(71.4)
4	Zygouris et al. (2017) [32]	A Preliminary Study on the Feasibility of Using a Virtual Reality Cognitive Training Application for Remote Detection of Mild Cognitive Impairment	*Journal of Alzheimer’s Disease*	Greece	Cohort study (pilot study)	Mild-Cognitive Impairment (MCI)	*N* = 12 MCI patients (*n* = 6) Healthy older adults (*n* = 6)	MCI patients: 64.5 ± 2.11 Healthy older adults: 63.0 ± 2.24	MCI patients: 2(33.3)/4(66.7) Healthy older adults: 1(16.7)/5(83.3)
5	Bonnechère et al. (2018) [33]	The Use of Mobile Games to Assess Cognitive Function of Elderly with and without Cognitive Impairment	*Journal of Alzheimer’s Disease*	Belgium	Cross-sectional design	Elderly with and without cognitive impairments	*N* = 76 Healthy young adults (*n* = 20) Old patients with Cognitive impairment (*n* = 29) Aged controls (*n* = 27)	Young adults: 26 ± 3 Old patients with cognitive impairment: 80 ± 12 aged controls: 74 ± 10	Unknown
6	Oh et al. (2018) [25]	Effects of smartphone-based memory training for older adults with subjective memory complaints: a randomized controlled trial	*Aging and Mental Health*	South Korea	Randomized controlled trial	Patients aged between their fifties and sixties who reported subjective memory complaints (SMC)	*N* = 53 EG (*n* = 18) CG1 (*n* = 19) CG2 (*n* = 16)	EG: 59.28 ± 5.11 CG1: 58.79 ± 5.00 CG2:59.949 ± 5.17	EG: 9(50)/9(50) CG1: 9(47.3)/10(52.7) CG2: 7(43.7)/9(56.3)
7	Elbogen et al. (2019) [26]	Cognitive Rehabilitation with Mobile Technology and Social Support for Veterans with TBI and PTSD: A Randomized Clinical Trial	*The Journal of Head Trauma Rehabilitation*	United States of America	Randomized controlled trial	Veterans aged between 18 and 65, meeting traumatic brain injury (TBI) and post-traumatic stress disorder (PTSD) criteria	*N* = 112 EG (*n* = 57) CG (*n* = 55)	All: 36.52 ± 8.42 EG: 36.77 ± 8.60 CG: 36.25 ± 8.30	All: 101(90)/11(10) EG: 52(90)/5(10) CG: 51(90)/4(10)
8	Powell et al. (2017) [34]	The development and evaluation of a web-based program to support problem-solving skills following brain injury	*Disability and Rehabilitation:* *Assistive Technology*	United States of America	Nonequivalent control group pretest–posttest design	Patients aged 18 years or older with post-brain injury and cognitive impairment	Group study *N* = 23 EG (*n* = 14) CG (*n* = 9) Single case study *N* = 1	EG: 40 ± 16(20–75) CG: 51 ± 8(33–64) Single case: 28	EG: 6(43)/8(57) CG: 5(56)/4(44) Single case: 0(0)/1(100)
9	Chudoba et al. (2020) [35]	The development of a manual-based digital memory notebook intervention with case study illustrations	*Neuropsychological Rehabilitation*	United States of America	Case study design	Individuals with cognitive impairment	*N* = 3	Varied in age from 39 to 72 years old	Unknown
10	Moon and Park (2020) [27]	The effect of digital reminiscence therapy on people with dementia: a pilot randomized controlled trial	*BMC Geriatrics*	South Korea	Randomized controlled trial	Moderate dementia, females ≥ 65 years old, registered at the daycare center	*N* = 49 EG (*n* = 24) CG (*n* = 25)	EG: 84.05 ± 6.23 CG: 82.96 ± 6.01	EG: 0(0)/25(100) CG: 0(0)/24(100)
11	Tsoy et al. (2020) [36]	Self-Administered Cognitive Testing by Older Adults at-risk for Cognitive Decline	*The Journal of Prevention of* *Alzheimer’s Disease*	United States of America	One-group pretest–posttest design	Older adults (over 70 years old) with high multimorbidity	*N* = 30	80 ± 6	Unknown
12	Robert et al. (2020) [28]	Efficacy of a Web App for Cognitive Training (MeMo) Regarding Cognitive and Behavioral Performance in People With Neurocognitive Disorders: Randomized Controlled Trial	*Journal of Medical Internet * *research*	France	Randomized controlled trial	Patients aged over 60 years old with mild or major neurocognitive disorders (NCDs)	*N* = 46 EG (*n* = 25) CG (*n* = 21)	All: 79.4 ± 6.8 EG: 79.8 ± 7.0 CG: 78.8 ± 6.6	All: 22(47)/24(52) EG: 15(60)/10(40) CG: 7(33)/14(66)
13	Oirschot et al. (2020) [37]	Symbol Digit Modalities Test Variant in a Smartphone App for Persons With Multiple Sclerosis: Validation Study	*JMIR mHealth and * *uHealth*	Netherlands	Cross-sectional study	Patients aged between 20 and 50 years with relapsing–remitting multiple sclerosis (MS)	*N* = 104 EG (*n* = 25): Relapsing-remitting MS CG (*n* = 79): Two groups of healthy control subjects	EG: 40.0 ± 8.0 CG 1: 37.0 ± 8.0 CG 2: 34.0 ± 8.0	EG: 2(8)/23(92) CG1: 4(19)/17(81) CG2: 29(50)/29(50)
14	Meltzer et al. (2021) [29]	Improvement in executive function for older adults through smartphone apps: A randomized clinical trial comparing language learning and brain training	*Aging, Neuropsychology, and Cognition,*	Canada	Randomized controlled trial	Healthy patients aged 65–75	*N* = 74 Duolingo group (*n* = 28) Brain HQ group (*n* = 24) Control group (*n* = 24)	Duolingo: 69.57 ± 2.97 Brain HQ: 70.08 ± 2.89 Control: 70.00 ± 2.62	Duolingo: 10(35.7)/18(64.3) Brain HQ: 7(29.2)/17(70.8) Control: 8(33.3)/16(66.7)
15	Bonnechère et al. (2021) [38]	Brain training using cognitive apps can improve cognitive performance and processing speed in older adults	*Scientific Reports*	United Kingdom	One-group pretest–posttest design	Healthy patients aged from 60 to over 80 years old	*N* = 12,000	Unknown	Unknown
16	Rosenblum et al. (2021) [39]	DailyCog: A Real-World Functional Cognitive Mobile Application for Evaluating Mild Cognitive Impairment (MCI) in Parkinson’s Disease	*Sensors*	Israel	Case study design	Parkinson’s disease patients with MCI, aged between 40 to 80 years	*N* = 36	Unknown	Unknown
17	Scullin et al. (2021) [30]	Using smartphone technology to improve prospective memory functioning: A randomized controlled trial	*Journal of the* *American Geriatrics Society*	United States of America	Randomized controlled trial	Older adults with cognitive impairment (mild cognitive impairment or mild dementia)	*N* = 48 EG (reminder app condition) = 23 CG (digital recorder app condition) = 25	EG (reminder app condition): 73.17 ± 6.00 CG (digital recorder app condition): 76.40 ± 8.02	EG: 11(48)/12(52) CG: 17(68)/8(32)
18	Weizenbaum et al. (2021) [40]	Smartphone-Based Neuropsychological Assessment in Parkinson’s Disease: Feasibility, Validity, and Contextually Driven Variability in Cognition	*Journal of the International Neuropsychological Society*	United States of America	Cohort study (quasi-longitudinal design)	Parkinson’s disease (PD) patients who were non-demented and had mild-to-moderate PD	*N* = 27	63.2 ± 8.7	14(51.9)/13(48.1)

### 3.4. Intervention Type, Outcome Measurements, and Main Results of Studies

Table 5 shows the type of intervention, the number, frequency, and duration of sessions, outcome measures, main results, and correlation or effectiveness in cognitive function for 18 studies. Five studies [31,33,36,37,40] were applied only once to evaluate cognitive function rather than actual intervention application. Eleven studies presented the contents of interventions for the comparison group [23,24,25,26,27,28,29,30,31,32,34], with one study [32] applying the same interventions to both the experimental and comparison groups.

In analyzing the number of sessions, frequency, and period, the most common study design consisted of one session, as five studies [31,33,36,37,40] focused on evaluating the possibility of using apps for cognitive function evaluation purposes. Four studies [27,30,32,35] had a duration of four weeks; two studies lasted eight weeks [24,25] and six months [23,36], and one study each lasted ten weeks [34], twelve weeks [28], and sixteen weeks [29]. One study [39] had an unknown period. The session duration ranged from the shortest at 15–20 min [25] to the longest at 90–120 min [35]. Many studies did not report the session duration.

Among the types of traditional neurological assessments, five studies [25,27,28,31,33] used the Mini-Mental State Examination (MMSE), an evaluation tool that screens overall cognitive function, and four studies [31,36,37,40] used evaluation apps. In addition, tools evaluating various cognitive functions (attention, memory, computational ability, problem-solving ability, executive function, etc.) were applied. Instrumental activities of daily living (IADL) [23,35], quality of life [30,35], depression [25,27], behavioral and psychological symptoms of dementia (BPSD) [27,28], participation [27], and game scores [33,38] were measured in addition to cognitive function.

Of the eighteen studies, sixteen showed positive clinical implications for the relationship or effectiveness of cognitive function due to the application of cognitive rehabilitation apps [23,24,25,28,29,30,31,32,33,34,35,36,37,38,39,40]. The remaining two studies [26,27] showed improvements in functions other than cognitive functions, such as emotional and behavioral control and depression. Still, they did not show significant differences in overall cognitive and executive functions.

Of the sixteen studies, four [31,36,37,40] demonstrated the availability of cognitive function evaluation using apps; two [33,38] showed a significant correlation between mobile game scores and cognitive function; and two [32,39] demonstrated that mild cognitive impairment (MCI) can be effectively identified through app usage results. The remaining eight studies [23,24,25,28,29,30,34,35] correlated with cognitive function improvement using apps for cognitive function training. The improved cognitive functions included reasoning and language ability, memory, concentration, information processing speed, and problem-solving ability.

**Table 5:** Intervention type, outcome measurements, and main results of studies.

No.	Author (Year)	Type of Intervention	Compare (or Control) Group Intervention	Session Number, Frequency, and Duration	Outcome Measurements	Results	Correlation or Effectiveness of Cognitive Function
1	Oliveir a et al. (2014) [31]	None [VR-based cognitive assessment with the Systemic Lisbon Battery (SLB): cognitive assessment]	Traditional neuropsychological evaluation with paper-and-pencil tests, along with a pilot version of the Systemic Lisbon Battery (SLB)	1 session (testing), 60 min	SLB: planning, memory, visuospatial ability, attention, working memory, and calculation. Traditional neuropsychological assessment: Wechsler Memory Scale, Rey Complex Figure, Cancelation Tests, Frontal Assessment Battery, Clock Drawing Test, Trail Making Test, and Mini-Mental State Examination	The SLB was able to identify the same cognitive deficits in stroke patients that traditional paper-and-pencil tests identify, and the pattern of correlations between the measures of both tests suggests that the SLB is a viable alternative to the traditional paper-and-pencil tests.	Validated
2	Corbett et al. (2015) [23]	Reasoning and problem-solving cognitive training (ReaCT), General cognitive training (GCT)	Internet-based tasks involving a game in which people were asked to put a series of statements in correct numerical order	10 min daily, 6 months (ReaCT intervention suggested completing 3 sessions each week)	Minimum Data Set-Home Care IADL, Reasoning, spatial working memory (SWM), and Digit vigilance (DV) tests	The ReaCT and GCT packages conferred significant benefits to reasoning and verbal learning at 6 months compared to controls. Both packages conferred significantly more significant benefits on the primary outcome measure of IADL than the control treatment at 6 months.	Validated
3	Pedullà et al. (2016) [24]	Adaptive cognitive rehabilitation (CR) intervention based on working memory (WM) exercises using the COGNI-TRAcK app (the difficulty level of the exercises was automatically adjusted based on the user’s performance)	Non-adaptive cognitive rehabilitation (CR) intervention based on working memory (WM) exercises using the COGNI-TRAcK app (the difficulty level of the exercises was kept constant throughout the intervention)	30-min sessions weekly for 8 weeks	Brief Repeatable Battery of Neuropsychological Tests (BRB-NT) and Wisconsin Card Sorting Test (WCST)	An adaptive workload in the COGNI-TRAcK intervention led to significant improvements in cognitive functions, including verbal memory acquisition, delayed recall, fluency, sustained attention, concentration, and information processing speed. These improvements were maintained even after 6 months, indicating the long-term effectiveness of the intervention.	Validated
4	Zygouris et al. (2017) [32]	Virtual Super Market (VSM) cognitive training exercise	Virtual Super Market (VSM) cognitive training exercise	At least once a day, 1 month	Time taken to complete the exercise and stores categorical measures related to the performance	Virtual Super Market (VSM) cognitive training application accurately detected mild cognitive impairment (MCI) in older adults. The average performance in duration to complete the exercise differed significantly between healthy older adults and those with MCI, yielding a correct classification rate of 91.8% with a sensitivity and specificity of 94% and 89%, respectively, for MCI detection.	Validated
5	Bonnechère et al. (2018) [33]	None (a set of seven short mobile games to assess cognitive functions)	Compare group (healthy young adults, old patients with cognitive impairment, aged control) received no intervention.	1 session (testing)	Scores obtained in seven mobile games, Mini-Mental State Exam (MMSE), and Addenbrooke’s Cognitive Evaluation revised (ACE-R)	Significant differences exist in all game scores between patients with cognitive impairments and aged controls. Significant correlations exist between the average game scores and the MMSE and ACE-R. (this suggests that cognitive mobile game scores could be used as an alternative to MMSE and ACE-R to evaluate cognitive function in older people with and without cognitive impairment, as long as the MMSE score is higher than 20/30)	Validated
6	Oh et al. (2018) [25]	Newly developed smartphone-based brain anti-aging and memory-reinforcement training (SMART) intervention	The control group was divided into two groups, i.e., an active control group that received Fit Brains training and a wait-list control group that did not receive any intervention during the study period.	40 sessions, 15–20 min of instruction per day, 5 days per week for 8 weeks	Korean version of the Mini-Mental State Exam (MMSE-K), Korean Wechsler Adult Intelligence Scale-IV (K-WAIS-IV), Memory Diagnostic System (MDS), Center for Epidemiologic Study-Depression (CES-D), State-Trait Anxiety Inventory (STAI), and Multifactorial Memory Questionnaire (MMQ)	An 8-week smartphone-based memory training program may improve working memory function in older adults. However, objective improvement in performance does not necessarily lead to decreased subjective memory complaints.	Validated
7	Elbogen et al. (2019) [26]	Cognitive Applications for Life Management (CALM), involving goal management training plus mobile devices for cueing and training attentional control	Brain Health Training involving psychoeducation plus mobile devices to train visual memory	6 months, 3 times (at 0, 2, and 4 months), 60–90 min	Delis-Kaplan Executive Function System (DKEFS), Barratt Impulsiveness Scale (BIS), Dimensions of Anger Reactions (DAR), Head Injury Behavior Scale (HIBS), and Clinician-Administered PTSD Scale (CAPS)	Cognitive Applications for Life Management (CALM) showed promise for improving emotional and behavioral regulation in veterans with co-occurring traumatic brain injury (TBI) and post-traumatic stress disorder (PTSD). There were no significant improvements in executive function.	The study did not provide clear evidence of effectiveness in cognitive function.
8	Powell et al. (2017) [34]	The Web-based program, ProSolv (uses a small number of coaching sessions to support problem-solving in everyday life)	Usual care condition: metacognitive strategy instruction without using an app for training problem-solving skills (uses a small number of coaching sessions to support problem-solving in everyday life)	20 to 45 min each week over 10 weeks	Group study: problem-solving Questionnaire (PSQ), Problem-Solving Rating Scale (PSRS), TBI Self-Efficacy Questionnaire (TBI-SE), Satisfaction with Life Scale (SWLS), and System Usability Scale (SUS) (ProSolv condition only) Single-case study: All group study outcome measurements, BRIEF, EMQ, and Structured role play	ProSolv program was associated with improvements in knowledge of problem-solving skills, higher ratings of problem-solving ability, and observed changes in problem-solving ability in a role-playing context.	Validated
9	Chudoba et al. (2020) [35]	A digital memory notebook (DMN) is designed to assist individuals with cognitive impairment in organizing daily tasks	There was no control group.	5–6 sessions, 90–120 min, 4 weeks (1 month)	Instrumental activities of daily living (IADLs), everyday memory difficulties, coping self-efficacy, and satisfaction with life	Two out of three participants self-reported a clinically significant reduction in memory lapses and improved daily functioning following the DMN intervention. All participants demonstrated clinically significant changes in coping with problems and building self-efficacy. All participants scored in the normative range post-intervention on satisfaction with life.	Validated
10	Moon and Park (2020) [27]	Digital reminiscence therapy (RT)	Storytelling sessions without digital materials	30 min/day, 2 times/week, 4 weeks	MMSE-DS, Cornell Scale for Depression in Dementia (CSDD), the Korean version of the neuropsychiatric inventory (K-NPI), and Engagement of a Person with Dementia Scale (EPWDS)	Depression was significantly decreased at T1 and T2 in the digital RT group compared to the control group. Engagement in the digital RT group was significantly increased at the last session compared to the control group. Cognition and BPSD were not significantly different between groups and time points.	The study did not provide clear evidence of effectiveness in cognitive function.
11	Tsoy et al. (2020) [36]	None (TabCAT: cognitive assessment)	There was no control group.	1 session (testing)	TabCAT: cognitive assessment included tests of executive functions and processing speed, Match, cognitive inhibition, Flanker, and spatial working memory (Stargazer)	Self-administered cognitive assessments in older adults at risk for cognitive decline are feasible and reliable. Consistency in cognitive scores was similar among participants with and without prior experience with touchscreen computing devices.	Validated
12	Robert et al. (2020) [28]	MeMo (Memory Motivation) Web app (1) active MeMo group: used MeMo about once every 2 days (2) nonactive MeMo group: used MeMo less	Treatment as usual, not MeMo	4 sessions/week, 30 min/session, 12 weeks	Mini-Mental State Examination (MMSE), Informant Questionnaire on Cognitive Decline in the Elderly (IQCODE), Frontal Assessment Battery (FAB), Free and Cued Selective Reminding Test (FCSRT), Trail Making Test A (TMT A), Stroop test, Digit Symbol Substitution Test (DSST), Neuropsychiatric Inventory (NPI), and Apathy Inventory (AI)	The active MeMo group showed improved cognitive and behavioral outcomes compared to participants in the nonactive MeMo group. Specifically, the active MeMo group demonstrated stable or improved performance in attention, executive functions, and apathy scores and higher performance levels in the MeMo app games compared to the nonactive MeMo group. (this suggests that regular use of the MeMo app positively impacts cognitive and behavioral outcomes in older adults with cognitive impairments.)	Validated (only with regular use of the app)
13	Oirschot et al. (2020) [37]	None (Symbol Digit Modalities Test (sSDMT): cognitive assessment)	There was no control group.	1 session (testing)	Smartphone variant of the Symbol Digit Modalities Test (sSDMT)	The sSDMT showed good construct validity and test–retest reliability compared to the paper-and-pencil SDMT. (the study suggests that the sSDMT is a valid and reliable tool for assessing cognitive processing speed in persons with MS and can potentially help monitor cognitive function in clinical practice.)	Validated
14	Meltzer et al. (2021) [29]	Duolingo group: Introductory Spanish course Brain HQ group: eight exercises focused on executive function, attention, and working memory, all tapping abilities	No specific intervention (passive control group)	30 min/day, 5 times/week for 16 weeks	N-back task, Simon task, and color–word interference task (Stroop task)	Both Duolingo language learning and BrainHQ brain training significantly improved executive function, and these improvements were significantly more significant than those observed in the control group.	Validated
15	Bonnechère et al. (2021) [38]	Cognitive Mobile Games (CMGs)	There was no control group.	100 sessions (vary between subjects and type of CMG)	Cognitive Mobile Games (CMG) score analyzed: seven games (Square Numbers, Memory Sweep, Word Pair, Babble Bots, Must Sort, Unique, and Rush Back)	The rate of improvement in Cognitive Mobile Games (CMG) performance is slower in older participants. Processing speed and CMG performance decrease with the increasing age of the participants.	Validated
16	Rosenblum et al. (2021) [39]	DailyCog app: smartphone application for the detection of mild cognitive impairment	There was no control group.	Unknown	Times that it took the users to perform tasks, Self-evaluation questionnaire (reflect the users’ feelings on how they view their cognitive state and their functional abilities)	The critical result of the study was the demonstration of the feasibility of using the DailyCog app for detecting and evaluating PD-MCI. The app effectively captured both stable and deteriorating patients with PD-MCI, indicating its potential for broader use in conditions involving cognitive decline.	Validated
17	Scullin et al. (2021) [30]	Cortana app (reminder app as their intervention)	Voice Recorder app (digital recorder app as their intervention)	4 weeks (no additional training sessions provided) (at first, one session of training on the general features of smartphones, which generally lasted 2–4 h)	Prospective memory performance was assessed twice each week by an interactive voice response system (Plum Fuse+)	Participants reported an overall improvement in their personally relevant prospective memory tasks, which were similar across the reminder app and digital recorder app conditions. The quality of life composite score also significantly improved from pre-to-post intervention.	Validated
18	Weizenbaum et al. (2021) [40]	None (MindLAMP Smartphone Assessments: cognitive assessment)	There was no control group.	1 session, 10-day assessment period	Individual difference questionnaires: Online self-report questionnaires related to trait mood, sleep, and executive function. In-lab assessment: UPDRS, MoCA, Trail Making Test, and WMS-III Spatial Span MindLAMP Smartphone Assessments: Brief survey of context, mood, alertness, motivation, caffeine, recent exercise, and medication ON–OFF state; Trails-B task and Backwards Spatial Span task	High response rate to prompts, demonstrating the feasibility of using smartphone assessments for cognitive performance evaluation in individuals with Parkinson’s disease. Strong convergent validity between traditional neuropsychological tests and smartphone working memory tests indicates smartphone assessment’s reliability. (these results support the feasibility, reliability, and validity of repeated smartphone assessments for cognitive evaluation in Parkinson’s disease, providing insights into the effects of context and individual factors on cognitive variability in this population.)	Validated

### 3.5. Mobile Applications Characteristics of Studies

Table 6 presents a comprehensive overview of the app’s name, platform type, main cognitive rehabilitation objective, cognition subdomain, occupational therapy intervention approach type, and language for eighteen studies. It is worth noting that a diverse range of app types were utilized across all eighteen studies. The platform type varied, with three studies [23,28,34] using web-based mobile apps on PC, smartphone, or tablet, and the remaining fifteen studies employing general mobile apps on Android or iOS platforms.

The main cognitive rehabilitation objective of the apps was cognitive function assessment in five studies [31,36,37,39,40] and cognitive function training in the remaining thirteen studies. In analyzing the subdomains of cognition, memory was addressed in seventeen studies [23,24,25,26,27,28,29,30,31,32,33,35,36,37,38,39,40], attention in eight studies [23,25,26,29,31,32,33,38], executive function in seven studies [26,29,32,33,36,39,40], speed of processing in five studies [28,29,36,37,38], and visuospatial ability in three studies [23,31,39].

Regarding the occupational therapy intervention approach types, the prevention approach was applied in all eighteen studies, the establishment and restoration approach was applied in fifteen studies [24,25,26,27,28,30,31,32,33,34,35,36,37,39,40], the maintenance approach in fourteen studies [23,24,25,26,27,28,29,30,32,33,34,35,38,39], and the creation and promotion approach in seven studies [23,29,31,36,37,38,40]. All studies applied one or more occupational therapy approaches.

As for the languages used, seven studies did not mention the language. Seven studies [26,28,29,34,36,38,39] used English, two [25,27] used Korean, and two [28,33] used French.

**Table 6:** Mobile application characteristics of studies.

No.	Author (Year)	App’s Name	Platform Type	The Main Cognitive Rehabilitation Object	Cognition Subdomain	Occupational Therapy Intervention Approach Type	Language
1	Oliveira et al. (2014) [31]	Systemic Lisbon Battery (SLB)	Tablet (Android)	Assessment	Planning, memory, visuospatial ability, attention, working memory, and calculation	Creation and promotion; establishment and restoration; and prevention	Unknown
2	Corbett et al. (2015) [23]	Unknown (reported as a ReaCT, GCT)	Web-based mobile app (PC, smartphone, or tablet)	Training	Reasoning and problem-solving, attention, memory, and visuospatial ability	Creation and promotion; maintenance; and prevention	Unknown
3	Pedullà et al. (2016) [24]	COGNI-TRAcK	Smartphone or tablet	Training	Working memory	Establishment and restoration; maintenance; and prevention	Unknown
4	Zygouris et al. (2017) [32]	Virtual Super Market (VSM)	Android (PC and web-based versions also exist)	Training	Visual and verbal memory, executive function, attention, and spatial navigation	Establishment and restoration; maintenance; and prevention	Unknown
5	Bonnechère et al. (2018) [33]	Peak	Smartphone or tablet	Training	Quantitative reasoning, arithmetic, working memory, attention, spatial memory, and executive functions	Establishment and restoration; maintenance; and prevention	French
6	Oh et al. (2018) [25]	Smartphone-based brain Anti-aging and memory Reinforcement Training (SMART)	Smartphone app (Android and iOS)	Training	Attention, memory, working memory, response inhibition	Establishment and restoration; maintenance; and prevention	Korean
7	Elbogen et al. (2019) [26]	Event Logger, Mind Jogger, IQ Boost	Mobile app	Training	Attention, working memory, and executive function	Establishment and restoration; maintenance; and prevention	English
8	Powell et al. (2017) [34]	ProSolv	Web-based mobile app	Training	Problem-solving	Establishment and restoration; maintenance; and prevention	English
9	Chudoba et al. (2020) [35]	Digital Memory Notebook (DMN)	Tablet with a keyboard attachment (iOS)	Training	Memory	Establishment and restoration; maintenance; and prevention	Unknown
10	Moon and Park (2020) [27]	Unknown (reported as a digital RT)	Smartphone app (android)	Training	Memory and recognition	Establishment and restoration; maintenance; and prevention	Korean
11	Tsoy et al. (2020) [36]	TabCAT	Tablet (iOS)	Assessment	Executive function, processing speed, cognitive inhibition, and spatial working memory	Creation and promotion; establishment and restoration; and prevention	English
12	Robert et al. (2020) [28]	MeMo (Memory Motivation)	Web-based mobile app	Training	Visual memory, working memory, associative memory, processing speed, inhibitory control, mental flexibility, and reaction anticipation	Establishment and restoration; maintenance; and prevention	French and English
13	Oirschot et al. (2020) [37]	MS sherpa	Smartphone app (Android and iOS)	Assessment	Cognitive processing speed	Creation and promotion; establishment and restoration; and prevention	Unknown
14	Meltzer et al. (2021) [29]	Duolingo, Brain HQ	Smartphone or tablet	Training	Processing speed, attention, memory, and executive functions	Creation and promotion; maintenance; and prevention	English
15	Bonnechère et al. (2021) [38]	Cognitive Mobile Games (CMG)	Smartphone or tablet (Android and iOS)	Training	Memory, reasoning, speed of process, and attention	Creation and promotion; maintenance; and prevention	English
16	Rosenblum et al. (2021) [39]	DailyCog	Smartphone app (android)	Assessment	Feelings about how they view their cognitive state (executive functions, visual–spatial abilities, and memory skills)	Establishment and restoration; maintenance; and prevention	English
17	Scullin et al. (2021) [30]	Unknown (two types of smartphone-based electronic memory aids: a reminder app and a digital recorder app)	Smartphone app (Android and iOS)	Training	Prospective memory	Establishment and restoration; maintenance; and prevention	Unknown
18	Weizenbaum et al. (2021) [40]	MindLAMP (Mind Learn–Assess–Manage–Prevent)	Smartphone or tablet (Android and iOS)	Assessment	Working memory and executive function	Creation and promotion; establishment and restoration; and prevention	Unknown

## 4. Discussion

Recently, the popularity of cognitive training mobile apps has been increasing, but there is a lack of evidence for their effectiveness as digital therapeutics. Therefore, this study systematically reviewed the literature on the application of mobile applications for cognitive rehabilitation and evaluated their effectiveness. As an intervention for cognitive rehabilitation, this study included various occupational therapy approaches such as creating and promoting, establishing and restoring, maintaining, preventing, and modifying. A study that measured cognitive function was included to verify the effectiveness of mobile app applications. Although general characteristics such as age and gender of the study subjects were not limited, subjects whose cognitive impairment was not the main symptom but had mental illness or behavioral disorder as the main symptoms were excluded for suitability for the study. Cognitive disorders caused by neurological diseases are due to structural or functional brain damage. These diseases have a precise pathological mechanism, and related studies can increase the validity and reliability of the study by maintaining a consistent target group. This is consistent with previous studies emphasizing the importance of research on cognitive impairment based on neurological mechanisms [41]. Mental illness and behavioral disorders are mainly associated with an imbalance of neurotransmitters or mental stress and have a different pathological mechanism than neurological diseases. Excluding these conditions avoids confusion in the research focus [42]. Mental illness and behavioral disorders can be accompanied by cognitive decline, but this is mainly considered a secondary phenomenon. By limiting the study subject group to cognitive impairment due to neurological disease, we tried to increase the reliability of the study results [43]. Additionally, the reason for including dementia and excluding schizophrenia is that dementia is a neurodegenerative disease with a direct pathological mechanism related to cognitive decline, while schizophrenia is a mental disease primarily associated with neurotransmitter imbalances and has different pathological mechanisms. By applying these criteria, we aimed to clarify the study’s focus and maintain consistency and reliability.

Eighteen studies were finally analyzed; eight randomized control experimental studies [23,24,25,26,27,28,29,30] used the PEDro scale, and ten non-randomized control experimental studies [31,32,33,34,35,36,37,38,39,40] used MINORS. The PEDro scale validates the methodological quality of clinical trials [44]. MINORS is the only valid quality assessment tool for non-randomized controlled trials [21]. Six [23,25,26,27,28,30] out of eight randomized control experimental studies met the quality criteria of Good, indicating that the study quality was high. In seven studies [23,24,25,26,28,29,30], excluding one [27], zero points were obtained in the therapist’s blind item, and most studies scored low overall in the subjects’, therapists’, and assistants’ blind items. This suggests that a blinded research design is required to improve the quality of future research. As a result of the quality evaluation of non-randomized control experimental studies using MINORS, three [32,36,37] out of ten met high quality criteria, and all seven met moderate quality criteria. Hence, the average quality evaluation results of the study were moderate. In particular, the overall low score was shown in the items of unbiased assessment of the study endpoint, follow-up period application to the aim of the study, and prospective calculation of the study size. To improve the quality of future research, blinding between the evaluator and the study subject is required when evaluating the measurement of research results. If blinding is impossible, the reason must be presented. Additionally, it is necessary to establish an appropriate follow-up observation period related to the research purpose and to plan the sample size sufficiently.

As a result of the general characteristic analysis of the eighteen studies, fourteen studies (77.77%) [25,26,27,28,29,30,33,34,35,36,37,38,39,40] were conducted over the past six years. Studies have been published in various journals, and the USA had the most research studies with six (33.33%) [26,30,34,35,36,40]. The target group included three studies [23,29,38] on healthy people, with study subjects’ samples as high as 12,000 [38] and 6742 [23]. The remaining studies involved subjects with cognitive disorders due to stroke, traumatic brain injury (TBI), mild cognitive impairment (MCI), multiple sclerosis (MS), and Parkinson’s disease (PD), with sample sizes ranging from 3 to 104. Most of the studies were conducted on older adults (40s to 80s). The study results show that processing speed and cognitive mobile game scores decrease as age increases [38], cognitive function decreases as age increases, and the possibility of accompanying neurological diseases increases. Hence, most studies have researched older adults. This result indicates that it is essential to distinguish between age-related changes and disorders according to cognitive conditions, such as MCI, when designing cognitive training apps. Five studies [31,33,36,37,40] used only one session for cognitive function evaluation. Four studies [27,30,32,35] lasted four weeks; two studies lasted eight weeks [24,25] and six months [23,36], and one study each lasted ten weeks [33], twelve weeks [28], and sixteen weeks [29]. The shortest application time of the intervention session was 15–20 min [25], and 90–120 min [35] was the longest. Many studies have not reported the application time. Healthy participants exhibited high frequency in intervention duration and session count, with 24 weeks [23], sixteen weeks [29], and 100 sessions [38]. For multiple sclerosis with cognitive impairment, the intervention duration was eight weeks [24], post-brain injury with cognitive impairment was ten weeks [34], and dementia was four weeks [27,30]. Comparing or generalizing research results is limited because the range of variables, such as the number of study subjects, study period, and session application time, is broad. Intervention duration is a crucial factor in determining the effectiveness of a study. Short intervention durations can be useful for analyzing the short-term effects of an app, but longer durations are necessary to confirm long-term effects. Intervention periods of more than six months have shown significant improvements in cognitive function [45,46]. Several studies have demonstrated the effectiveness of cognitive rehabilitation apps in improving cognitive function in patients with mild cognitive impairment (MCI). According to a Cochrane review [47], at least twelve weeks of computer-based cognitive rehabilitation positively impact the maintenance and improvement of cognitive function. Long-term studies, such as those confirming the maintenance of effects twelve months post-intervention, are not just necessary but are significant in analyzing the effectiveness of cognitive rehabilitation apps [48].

Of the eighteen studies, fifteen used general mobile apps (smartphone or tablet), and the remaining three [23,28,34] used web-based mobile apps (PC, smartphone, or tablet). The main purpose of applying the cognitive rehabilitation apps analyzed was that thirteen (72.23%) studies focused on cognitive function training, and the remaining five (27.77%) [31,36,37,39,40] focused on cognitive function assessments. As a subdomain for cognitive function training or evaluation, memory showed the highest proportion with seventeen (94.44%) studies, followed by attention, executive function, speed of processing, listening, and visuospatial ability. Outcome measurements were most common, with five (27.77%) [25,27,28,31,33] studies applying the Mini-Mental State Examination (MMSE), a tool for screening overall cognitive function, followed by four (22.22%) [31,36,37,40] studies applying evaluation apps. In addition, various neurological evaluation tools were applied to evaluate cognitive function. According to the purpose of the study, instrumental activities of daily living (IADL), quality of life, depression, participation, behavioral and psychological symptoms of dementia (BPSD), and game scores were measured, showing significant differences [23,27,28,30,38]. These results indicate the applicability of these indicators and cognitive function as indices to measure the effectiveness of intervention applications for cognitive rehabilitation. They will provide helpful information for future related research. This study categorized the characteristics of apps based on the titles and contents listed in the papers, sorting them by app name, platform type, application purpose, cognitive domain, intervention type, and language. The Mobile Application Rating Scale (MARS) is a widely used mHealth app quality assessment tool that includes multidimensional measurements of not only subjective app quality but also engagement, functionality, esthetics, and information [49,50]. To apply the Mobile Application Rating Scale (MARS), it is necessary to directly execute and use the apps to evaluate detailed aspects such as engagement, functionality, esthetics, information, subjective quality, and app-specific items. However, there were significant limitations in directly executing the apps based solely on the information provided in the papers. Some studies provided partial screenshots, but issues such as the inability to find the apps in searches, improper execution, and lack of detailed program names were prevalent. Future studies aim to overcome data collection limitations based on papers by directly searching for and evaluating commercially available apps on platforms like the Google Play Store and Apple App Store using MARS. This approach is expected to enhance the reliability and validity of the MARS evaluations. Consequently, adopting this method will improve the quality of the research and provide more precise insights into the effectiveness and quality of mHealth apps.

As a result of applying the cognitive rehabilitation app, sixteen studies (88.88%) [23,24,25,28,29,30,31,32,33,34,35,36,37,38,39,40] showed positive clinical implications for the relationship or effectiveness of cognitive function. Among them, eight studies [23,24,25,28,29,30,34,35] demonstrated the correlation between using apps for cognitive function training and improving cognitive function. The main research results showed that the group that applied cognitive rehabilitation apps improved reasoning, language learning ability, and IADL performance [23] and showed improvement in verbal memory, delayed recall, fluency, concentration, and information processing speed, with long-term effects maintained even after six months [24]. Additional improvements included problem-solving ability [34], working memory [25], IADL and daily memory, self-efficacy and life satisfaction [35], attention, executive function [28], executive function [29], prospective memory, and quality of life [30]. Other studies have suggested that cognitive rehabilitation using mobile health apps can be effective for the elderly and patients with neurodegenerative diseases. In the study of Vaportzis et al. [51], the acceptability and usefulness of tablet training were high for the elderly. The study by Bier et al. [52] reported that even patients with cognitive impairment could apply the skills acquired during training to other smartphone and tablet functions in their daily lives. Four studies [31,36,37,40] demonstrated the possibility of using cognitive function evaluation using apps, and two studies [33,38] showed a significant correlation between mobile game scores and cognitive function. Two studies [32,39] demonstrated that mild cognitive impairment (MCI) could be effectively screened through the results of using apps.

On the other hand, two studies [26,27] did not show significant results in the effectiveness of applying mobile apps for cognitive rehabilitation. One [26] used veterans with TBI and PTSD symptoms, showing the possibility of improving emotional and behavioral control but not executive function. The other [27] applied digital reminiscence therapy to the elderly with dementia, which reduced depression and increased participation but showed no significant difference in cognitive functions and behavioral and psychological symptoms of dementia (BPSD). However, these results suggest that cognitive rehabilitation interventions for cognitive disabilities can consider not only cognitive function but also improving functions such as emotional and behavioral control, depression reduction, and participation. Cognitive dysfunction exhibits differentiated characteristics according to the general characteristics and the characteristics of the subject’s disease, so the response to the cognitive training intervention will also differ [53]. Therefore, it will be challenging to draw an accurate conclusion on the effectiveness of mobile apps in solving specific cognitive impairments if these differences are not considered.

According to the Occupational Therapy Practice Framework: Domain and Process (4th ed.) document published by the American Occupational Therapy Association [54], the occupational therapy intervention approach type of the cognitive rehabilitation mobile app used in the study was analyzed. All eighteen studies (100%) applied the prevention approach; fifteen studies (83.33%) [24,25,26,27,28,30,31,32,33,34,35,36,37,39,40] applied the establishment and restoration approach; and fourteen studies (77.77%) [23,24,25,26,27,28,29,30,32,33,34,35,38,39] applied the creation and promotion approach. It is thought that all studies applied the prevention approach because cognitive rehabilitation aims to prevent cognitive dysfunction and deterioration. Various studies have proven that cognitive rehabilitation apps are effective in improving cognitive function across diverse populations, suggesting that they may help prevent cognitive impairments. However, to strongly support these claims, long-term studies and additional clinical trials are required. The establishment and restoration approaches were likely prevalent due to the characteristics of the study subjects. Most studies targeted older adults with cognitive impairments due to neurological defects, so the app was used to establish and restore these cognitive impairments and prevent further deterioration. Additionally, since the mobile app was applied to evaluate cognitive function or verify the effectiveness of cognitive training in healthy adults, the creation and promotion approach was also applied [45]. Cognitive rehabilitation apps can be effective in maintaining and improving cognitive function, but it is essential to manage side effects such as fatigue and addiction. Various studies suggest the following strategies to manage these factors effectively: It is essential to take adequate breaks between cognitive rehabilitation sessions and incorporate physical activity to reduce fatigue. Research indicates that physical activity can help reduce fatigue and improve cognitive function [55]. Limiting the usage time of cognitive rehabilitation apps can prevent addiction; for instance, setting a limit on usage time per day is recommended [56]. Monitoring the user’s fatigue levels and usage patterns to create a personalized rehabilitation plan is effective. Cognitive rehabilitation professionals, such as occupational therapists, can support this approach by adjusting the rehabilitation activities to appropriate difficulty and frequency levels based on each individual’s needs.

The app’s target users may include individuals with Parkinson’s disease, mild cognitive impairment (MCI), vascular dementia, Alzheimer’s disease, cognitive impairment due to traumatic brain injury (TBI), and cognitive impairment due to multiple sclerosis (MS). It is essential to distinguish between these types of cognitive impairments associated with each condition and actively conduct research measuring the effectiveness of cognitive rehabilitation apps tailored to the characteristics of each disease. A systematic review and meta-analysis study on the effectiveness of cognitive rehabilitation programs for patients with Parkinson’s disease [57] showed positive improvements in attention, working memory, verbal memory, executive function, and processing speed. The meta-analysis revealed moderate effects on overall cognitive status and working memory and minor effects on verbal memory, overall cognitive function, and executive function. However, minor effects were observed in attention, visual memory, verbal fluency, and processing speed, and no effects were found in visuospatial abilities. According to the study, these rehabilitation programs can improve cognitive skills in the short term, but the sustainability of these improvements remains unclear. MCI is an intermediate stage between normal aging and dementia, with memory decline as the primary symptom. MCI patients are at a higher risk of progressing to Alzheimer’s disease, making early intervention crucial to maintaining or improving cognitive function. Cognitive rehabilitation programs for MCI patients have shown effectiveness in improving attention, memory, and executive function [58]. These programs can help maintain cognitive function and slow the progression of Alzheimer’s disease in the long term. Alzheimer’s disease is a slowly progressing degenerative brain disorder where continuous cognitive stimulation is essential. Cognitive rehabilitation apps can provide regular and long-term cognitive stimulation, helping slow cognitive function decline. A meta-analysis of various clinical trials [59] concluded that cognitive rehabilitation improves daily functioning and quality of life in Alzheimer’s patients. This analysis focused on improving overall cognitive function and task performance levels rather than specific cognitive functions. A pilot study targeting the Irish population [60] showed the positive impacts of cognitive rehabilitation on maintaining and enhancing cognitive function in early-stage Alzheimer’s patients. The study found significant improvements in memory, attention, and executive function among patients who underwent cognitive rehabilitation. A review study [61] analyzed how various cognitive rehabilitation approaches contribute to the recovery of cognitive function and the enhancement of quality of life in TBI patients. The study emphasized improvements in major cognitive domains such as attention, memory, and executive function. Research utilizing computer-based cognitive rehabilitation programs for MS patients [55] showed significant improvements in cognitive function, particularly in attention and memory. These programs helped reduce the cognitive difficulties patients experienced in their daily lives. Based on the analysis of the effectiveness of cognitive rehabilitation applied to various diseases described above, further active research is needed to analyze the effectiveness of apps as digital therapeutics for cognitive rehabilitation.

## 5. Conclusions

This study systematically reviewed the literature on the use of mobile applications for cognitive rehabilitation and evaluated their effectiveness. Despite the growing interest in cognitive rehabilitation mobile applications, the evidence supporting their effectiveness remains limited. The broad range of variables, such as the number of study subjects, study duration, and session application time, presents challenges in generalizing and standardizing research findings.

To address these limitations, future research should focus on conducting well-designed randomized controlled trials (RCTs) that encompass diverse characteristics of cognitive impairment subjects and ensure sufficient sample sizes for statistically significant results. Additionally, long-term studies with appropriate follow-up periods are essential to verifying the sustained effectiveness of these interventions.

This study holds clinical significance by demonstrating the effectiveness of cognitive rehabilitation mobile applications from an occupational therapy perspective. The primary aim of cognitive rehabilitation—assessing the presence or absence of cognitive impairment—was evaluated early in the intervention process. The applications were utilized to prevent and maintain cognitive function in healthy adults and to treat, maintain, and prevent further deterioration in individuals with cognitive impairments.

Our findings highlight the potential for cognitive rehabilitation mobile applications to be seamlessly integrated into daily life, thereby enhancing individual quality of life through prevention and treatment. These applications can increase accessibility to healthcare and ensure continuous expert management and support. By illustrating the strong connection between cognitive rehabilitation mobile applications and everyday activities, this study underscores the importance of these tools in real-life settings.

In conclusion, while this study provides promising evidence for the use of mobile applications in cognitive rehabilitation, further rigorous research is necessary to establish their efficacy and optimize their application. Long-term, well-structured studies are crucial to solidifying the role of cognitive rehabilitation mobile applications as effective digital therapeutics.

## Figures and Tables

**Table 1:** Keywords related to cognition, mobile app-based cognitive rehabilitation, cognitive function.

Domain	Keywords
Cognition disorder	(“cognition disorders”[MeSH] OR “cognitive dysfunction”[MeSH] OR “cognitive defect”[Emtree] OR “cognitive-impairment”[TIAB])
Mobile application-based cognitive rehabilitation	(“mobile applications”[MeSH][Emtree] OR “mobile Apps”[TIAB] OR “smartphone apps”[TIAB] OR “apps”[TIAB]) AND (“cognitive rehabilitation”[MeSH] OR [(“cognition”[MeSH][Emtree] OR “cognitive function”[TIAB]) AND(“rehabilitation”[MeSH] OR “therapy”[TIAB] OR “remediation”[TIAB] OR “training”[TIAB])]
Cognitive function	“cognition” OR “cognitive function”

## Data Availability

This article includes all the data supporting the reported results; further details on the methodology are available from the corresponding author upon reasonable request.
